# Forecasting effects of transport infrastructure on endangered tigers: a tool for conservation planning

**DOI:** 10.7717/peerj.13472

**Published:** 2022-05-17

**Authors:** Neil H. Carter, Narendra Pradhan, Krishna Hengaju, Chinmay Sonawane, Abigail H. Sage, Volker Grimm

**Affiliations:** 1 University of Michigan, Ann Arbor, United States of America; 2 International Union for Conservation of Nature, Kathmandu, Nepal; 3 Stanford University, Stanford, United States of America; 4 US Fish and Wildlife Service, Wenatchee, United States of America; 5 Helmholtz Centre for Environmental Research –UFZ, Leipzig, Germany

**Keywords:** Agent-based model, Carnivore, Conservation, Railway, Road, Protected area, Infrastructure

## Abstract

The rapid development of transport infrastructure is a major threat to endangered species worldwide. Roads and railways can increase animal mortality, fragment habitats, and exacerbate other threats to biodiversity. Predictive models that forecast the future impacts to endangered species can guide land-use planning in ways that proactively reduce the negative effects of transport infrastructure. Agent-based models are well suited for predictive scenario testing, yet their application to endangered species conservation is rare. Here, we developed a spatially explicit, agent-based model to forecast the effects of transport infrastructure on an isolated tiger (*Panthera tigris*) population in Nepal’s Chitwan National Park—a global biodiversity hotspot. Specifically, our model evaluated the independent and interactive effects of two mechanisms by which transport infrastructure may affect tigers: (a) increasing tiger mortality, *e.g.*, via collisions with vehicles, and (b) depleting prey near infrastructure. We projected potential impacts on tiger population dynamics based on the: (i) existing transportation network in and near the park, and (ii) the inclusion of a proposed railway intersecting through the park’s buffer zone. Our model predicted that existing roads would kill 46 tigers over 20 years via increased mortality, and reduced the adult tiger population by 39% (133 to 81). Adding the proposed railway directly killed 10 more tigers over those 20 years; deaths that reduced the overall tiger population by 30 more individuals (81 to 51). Road-induced mortality also decreased the proportion of time a tiger occupied a given site by 5 years in the 20-year simulation. Interestingly, we found that transportation-induced depletion of prey decreased tiger occupancy by nearly 20% in sites close to roads and the railway, thereby reducing tiger exposure to transportation-induced mortality. The results of our model constitute a strong argument for taking into account prey distributions into the planning of roads and railways. Our model can promote tiger-friendly transportation development, for example, by improving Environmental Impact Assessments, identifying “no go” zones where transport infrastructure should be prohibited, and recommending alternative placement of roads and railways.

## Introduction

Transportation networks are rapidly proliferating in some of the most biodiverse regions on earth (Alamgir et al., 2017). Improved transportation networks can provide important benefits to human societies, but can also harm wildlife populations and biodiversity (Laurance et al., 2014). Roads can fragment habitats and act as barriers to animal movement, thereby reducing gene flow and population connectivity (Westekemper et al., 2021). Roads can increase animal mortality due to collisions with vehicles and increase hunting pressure by facilitating human access into remote areas (Clements et al., 2014). Moreover, new roads portend increasing human disturbances from settlement growth and traffic noise and lights (Fahrig & Rytwinski, 2009). Although studies have elucidated a range of road impacts on wildlife (Van Der Ree, Smith & Grilo, 2015), in most cases little is known about the consequences of roads on wildlife population dynamics and persistence (Kramer-Schadt et al., 2004; Jaeger et al., 2005). This gap is especially large for endangered species despite the likelihood that transport infrastructure could increase their extinction risks (Beaudry, de Maynadier Jr & Hunter, 2008; Schmidt, Lewison & Swarts, 2020). Helping fill this gap can therefore better inform transportation planners to reduce negative impacts of these projects on endangered wildlife. This information would be especially useful during the early stages of infrastructure planning, prior to construction, to safeguard species of concern before it is too late (Quintero et al., 2010).

Road development is a major threat to the globally endangered tiger (*Panthera tigris*), a conservation umbrella species (WWF International, Dalberg, 2016). Thanks to conservation efforts, the global tiger population has increased over the last decade to just under 5,000 animals (Jhala et al., 2021). However, tiger habitat continues to be lost across their 13-country range (Joshi et al., 2016), and that loss may increase with expanding transportation networks across Asia. A recent study found that 134,000 km of roads already exist within tiger range and 24,000 km of new roads will be added by 2050 (Carter et al., 2020). Many of these roads do and will traverse protected areas, including national parks and tiger reserves, which are often source populations of tigers (Wikramanayake et al., 2011; Carter, Baeza & Magliocca, 2020; Quintana et al., 2022). One vital source population is Nepal’s Chitwan National Park—harboring approximately 100 adult tigers (Walston et al., 2010; DNPWC, 2018). The park is a UNESCO World Heritage Site and a global priority for tiger conservation (Sanderson et al., 2010). The park is also undergoing growth in transportation networks (MoFSC, 2015; Sharma et al., 2018). In 2016, the International Union for Conservation of Nature (IUCN) identified transport infrastructure as a major threat to the park’s “Outstanding Universal Value” (Van Merm & Talukdar, 2016). Moreover, the limited research on the impacts of transport infrastructure indicates that the expansion of roads and railways could come at the expense of the Chitwan tiger population. In Russia, vehicle collisions caused 1 in every 12 deaths of tigers monitored from 1992 to 2005 (Goodrich et al., 2008). In China and Sumatra, tiger and tiger prey occupancies decreased with increasing proximity to roads (Linkie et al., 2006; Wang et al., 2018). Therefore, roads may be shrinking the availability of suitable habitat for tigers and reducing landscape carrying capacities for tigers as a result (Chapman & Byron, 2018). These previous studies provide crucial insights, yet we still know little about how the configuration of roads and railways impact the persistence of tiger populations over time. In addition, we do not have a mechanistic understanding of how transport infrastructure affects tiger population dynamics.

Predictive models that forecast the future impacts to tigers can guide land-use planning in ways that proactively reduce the negative effects of transport infrastructure (Carter, Baeza & Magliocca, 2020). For example, land-use planners can adjust the proposed alignment of roads or railways to avoid high priority habitats or wildlife populations that are predicted to be at risk from transport infrastructure (Quintero et al., 2010). They can also better locate wildlife crossing structures to facilitate dispersal (Malo, Suárez & Díez, 2004). Agent-based models (ABM) are well suited for predictive scenario testing of this sort (McLane et al., 2011; Schulze et al., 2017). They enable the control for and experimental manipulation of sources of variability in the environment, including future changes such as proposed transport infrastructure or development (Schulze et al., 2017). Furthermore, ABMs are spatially explicit and based on individual behaviors, and can therefore provide a mechanistic understanding of how the effects of transportation projects ramify from individual to population levels (McLane et al., 2011; Carter, Baeza & Magliocca, 2020). Previous studies have used ABMs to evaluate measures for reducing collisions between vehicles and wildlife and to examine how roads block animal movements and distributions (Rupp & Rupp, 2010; Watkins et al., 2015; Barbosa et al., 2020). However, ABMs that examine the effects of transport infrastructure on animal population dynamics are rare (Ascensão et al., 2013; Barbosa et al., 2020) and none to our knowledge have been applied to endangered species. Furthermore, ABMs have not explored how roads alter predator populations via their effects on prey populations.

To help fill that gap, we build on an existing ABM of tiger territories in Chitwan National Park to simulate different effects and predict the future impacts of transport infrastructure on tiger population dynamics. Previously the ABM was used to link tiger territory dynamics with population dynamics (Carter et al., 2015), as well as to examine how prey depletion by nearby human settlements affect tiger populations (Carter, Levin & Grimm, 2019). Model outputs were shown to match a number of observed patterns in tiger behavior and population dynamics (Carter et al., 2015; Carter, Levin & Grimm, 2019). Here, we extend the model by testing the independent and interactive effects of two mechanisms by which transport infrastructure may affect tigers. One mechanism is through direct mortality (*e.g.*, vehicle and train collisions) associated with roads and railways. The second mechanism is through the depletion of mammal prey abundances near roads and railways. We chose these two mechanisms because studies show these transportation-induced impacts on mammals are widespread and empirical estimates exist in the literature to parameterize the model (Benítez-López, Alkemade & Verweij, 2010; González-Gallina et al., 2013). We had three objectives: (1) implement the two different mechanisms by which transport infrastructure affects tigers; (2) evaluate the impacts to the Chitwan tiger population over the next 20 years based on the existing transportation network in and near the park; and (3) evaluate the future impacts on the tiger population of the proposed railway alignment through the park’s buffer zone. We predict that transportation-induced mortality will significantly decrease the tiger population over 20 years when compared to a baseline without transportation-induced mortality, and that these lethal impacts will be amplified when prey numbers are also decreased.

## Materials and Methods

### Study area

**Figure 1:**
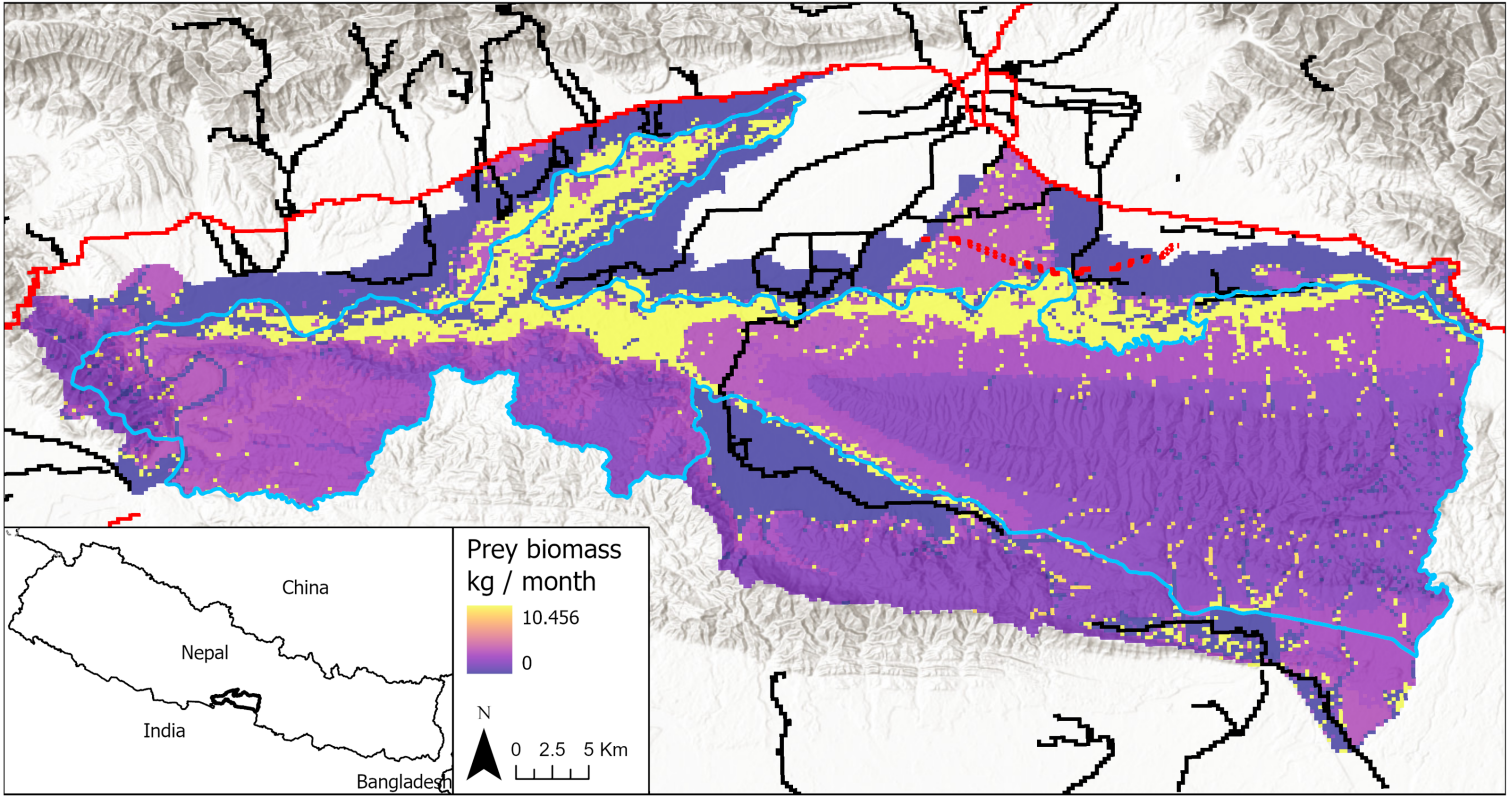
Study area in Chitwan, Nepal (83.8E, 27.7N; 84.8E, 27.3N), including both the national park and its buffer zone. Park boundary in blue. Primary roads are shown in red and secondary roads in black. The proposed railway alignment through the park’s buffer zone is shown in red dash marks. Prey biomass production is shown for each cell in the model landscape, with darker colors being low prey resources and lighter colors being high prey resources, including yellow indicating grid cells with highest estimated prey abundances.

We applied our model to Chitwan National Park, situated in the Himalayan lowlands in south central Nepal (Fig. 1, Sharma, 1990). The park was established in 1973 and is approximately 950 km^2^. It is located in a river valley basin along the flood plains of the Rapti, Reu, and Narayani Rivers with an elevation range of 150–815 m. A buffer zone surrounding the park was established in 1996 and is about 750 km^2^. The park and buffer zone consist of natural vegetation communities including sal (*Shorea robusta*) forest, khair (*Acacia catechu*) and sissoo (*Dalbergia sissoo*) riverine forests, and grasslands dominated by species of the genera *Saccharum*, *Themeda*, and *Imperata* (Chaudhary, 1998; Carter et al., 2013). These vegetation types in the park support a number of large herbivorous species, including spotted deer (*Axis axis*), barking deer (*Muntiacus muntjak*), wild boar (*Sus scrofa*), sambar (*Rusa unicolor*), hog deer (*Axis porcinus*) and gaur (*Bos gaurus*) (Seidensticker & McDougal, 1993)—all of which tigers prey upon. Other large predators in the park include the common leopard (*Panthera pardus*) and dhole (*Cuon alpinus*). The climate in the park is subtropical with a cool dry winter and a summer monsoon season from mid-June to late-September.

Many existing roads in the lowlands region are going to be upgraded and expanded in the near future to support greater traffic volumes and speeds (NPC, 2020). For example, the 1,795 km long Postal (Hulaki) highway—oldest highway in Nepal—is being resurfaced. The 1,028 km East-West (Mahendra) highway is being expanded from two to four lanes. Both of these highways pass through forests, habitat corridors, and protected areas including Chitwan (Van Merm & Talukdar, 2016; Quintana et al., 2022). Development and expansion of municipal roads that pass through Chitwan—such as the Thori-Madi-Bharatpur road—are also ongoing or proposed (Van Merm & Talukdar, 2016). Furthermore, the Government of Nepal is planning cross-country railway projects, including the 1,205 km long East-West electrified railway (NPC, 2020). Construction of this railway has begun in eastern Nepal. Currently, the railway is projected to intersect the Barandabhar corridor in Chitwan’s buffer zone (Fig. 1); however, the exact alignment and design elements through Barandabhar and other sensitive forested areas are still under discussion.

### Model parameterization

A long-term study began in the park core in the 1970s to collect field measures on many aspects of tiger behavior and ecology, including interactions with conspecifics and their environment, as well as population vital rates, such as survival and mortality (Sunquist, 1981; Smith, McDougal & Sunquist, 1987; Smith, 1993; Kenney et al., 2014). Those extensive field measures supplemented with those from other studies were directly used as parameters in our model (Table 1) instead of fitting the full model to data, that is, by calibration. In previous studies (Carter et al., 2015; Carter, Levin & Grimm, 2019), we conducted a sensitivity analysis and assessed model fit using a pattern-oriented modeling approach (Grimm et al., 2005), which compares multiple patterns observed in real systems to those generated by the model. Through those previous analyses, we determined that a range of key model outputs, such as reproduction rates, dispersal distances, resource selection, and land tenure, matched closely with observed patterns of the real tiger population in the park (Carter et al., 2015; Carter, Levin & Grimm, 2019). Furthermore, the overall response of the position and size of territories to different prey and tiger densities was realistic (Carter, Levin & Grimm, 2019). We are therefore confident that the model performs well and serves as an excellent foundation to address our central research question about the future impacts of transportation networks on tigers. In the following, we outline major components of the model but direct readers to Carter et al. (2015) which includes a detailed description of the model following the Overview, Design concepts, and Details (ODD) protocol (Grimm et al., 2006; Grimm et al., 2010) for describing ABMs.

**Table 1:** Summary of parameter information used in agent-based model of tiger populations in Chitwan National Park, Nepal.

**Parameters**	**Values**	**Reference**	**Notes**
Age-classes		Karanth & Stith, 1999 (Page 103)	Based on long-term field data of tigers across sites.
Breeding	3+ years old		
Transient	2–3 years old		
Juvenile	1–2 years old		
Cub	0–1 years old		
Litter size distribution^a^		Kenney et al., 2014 (Appendix A)	Based on long-term field data of tigers in Chitwan.
1	0		
2	0.23		
3	0.58		
4	0.17		
5	0.02		
Maximum number of cells female can add to territory per time step^a^	48 (3 km^2^)	Sunquist, 1981 (Derived from table 15 on page 37)	This value represents an approximation of the average area added to female’s territory per month from observed data.
Annual survival^a^^,^^b^		Karanth & Stith, 1999 (Page 103)	Survival rates were parameterized from field data on tigers, leopards, and cougars.
Breeding male	0.8		
Breeding female	0.9		
Dispersal male	0.65		
Transient male	0.65		
Transient female	0.7		
Juvenile	0.9		
Cub	0.6		
Annual fecundity^a^		Kenney et al., 2014 (Appendix A)	Based on long-term field data of tigers in Chitwan.
Probability that 3-year old resident female breeds	0.9		
Probability that 4+ year old resident female breeds	1		
Maximum possible dispersal distance from natal range^a^		Smith, 1993 (Table 1 on page 173)	Based on long-term field data of tigers in Chitwan.
Transient male	66 km		
Transient female	33 km		
Prey thresholds^a^			
Minimum within territory	76 kg/month	Miller et al., 2014 (Page 127)	Model estimates 2.5 kg/day to maintain basal metabolic rate of female Bengal tiger in Bangladesh. This converts to: (2.5 kg/day * 365 days)/12 months
Maximum within territory	167.3/month	Sunquist, 1981 (Page 91)	From empirical data, estimates female tiger in Chitwan consumes 5–6 kg/day. This converts to: (5.5 kg/day * 365 days)/12 months
Probability that dominant female will take territory patch from subordinate female if patch has highest prey^a^	0.25	Carter et al., 2015	Based on expert opinion.
Proportion of prey within territory utilized by female tiger^a^	0.1	Karanth et al., 2004 (page 4854)	Based on field data of large carnivore guilds across different sites in Asia and Africa.
Radius in which breeding males will search for nearby breeding females^a^	3 km	Ahearn et al., 2001 (Table 1 on page 90)	Based on long-term field data of tigers in Chitwan.
Max number of female territories a male can overlap^a^	6	Kenney et al., 2014 (Appendix A)	Based on long-term field data of tigers in Chitwan.
Litter sex ratio at birth	50:50	Karanth & Stith, 1999 (Page 103)	Based on long-term field data of tigers across sites.
Gestation period	3 or 4 months with equal probability	Sunquist, Karanth & Sunquist, 1999 (Page 7)	Gestation is 103 days, which is between 3 and 4 months. Model randomly selects either 3 or 4 months.
Search criteria for dispersing females to determine location of territory origin^a^		Carter et al., 2015	Based on expert opinion.
Ideal area in which no other female territory occurs	12.57 km^2^(2 km radius)		
Less-optimal area in which no other female territory occurs	3.14 km^2^(1 km radius)		
Probability that the transient male dies during challenge^a^	0.25	Kenney et al., 2014 (Appendix A)	Based on long-term field data of tigers in Chitwan.
Probability that the breeding male dies during challenge^a^	0.6	Kenney et al., 2014 (Appendix A)	Based on long-term field data of tigers in Chitwan.
Probability offspring die due to infanticide following successful challenge^a^		Pusey & Packer, 1994 (derived from Figure 1 on page 279)	Based on long-term field data on African lions in Tanzania’s Serengeti National Park.
Juvenile	0.24		
Cub	0.79		

**Notes.**

The model was based on data collected largely in Nepal’s Chitwan National Park.

a Parameters that were included in sensitivity analysis, described in Carter et al., 2015.

b Survival rates of adult females were reduced when intersected by primary or secondary roads, or the proposed railway. Main text has details.

### Mapping prey biomass and transport infrastructure

Previous research in the Nepal lowlands used a Poisson regression to relate land cover to tiger prey abundance and then mapped the fitted abundance estimates across the region at a resolution of 250 × 250 m (Shrestha, 2004). As our model time step was one month, we converted the abundance estimates to a monthly rate of prey biomass production per cell (0–10.46 kg per month, average = 3.78 kg per month) by scaling the abundance estimates to empirical rates of average daily prey consumption by females and information on female territory sizes in Chitwan (Table 1; Fig. 1). We simplified the model by holding prey biomass in each cell constant over time. Although seasonal change and predation can affect prey biomass over time (Karanth et al., 2004), we ignore those dynamics here to focus instead on transportation-induced changes in prey density.

We defined a road as “primary” if it was maintained year-round and provided access between large towns and “secondary” if it was not regularly maintained but allowed access between villages or into forested lands. Primary roads were paved, allowing for high-speed traffic, whereas secondary roads were either paved or hard-packed dirt. We collected existing data on the geographic locations of roads in Chitwan from OpenStreetMap, which provided the most comprehensive and accurate road data. Specifically, we used the R package *osmdata* to select roads in Chitwan and assign them as primary or secondary roads based on OpenStreetMap’s tags and our knowledge of the area. Roads with key “highway” and values “motorway” were classified as primary roads. Those with values “primary”, “secondary”, or “tertiary” were classified as secondary roads. Finally, the location (as of 2018) of the proposed railway through Barandabhar forest corridor was digitized from hardcopy maps provided by the Nepal Department of Transportation. Most recent plans indicate the proposed railway will still be constructed through Barandabhar forest, though the exact location is uncertain. We assumed the railway has the same effect on tigers as a primary road. We chose this assumption because railways are major linear infrastructure; however, we lacked empirical data to differentiate effects of railways from primary roads. In total, there were approximately 198 km of primary roads including the railway and 723 km of secondary roads in the model landscape.

### Simulating tigers and their territories

In the model, tigers follow a full life cycle—born, grow, reproduce, disperse, establish and modify territories, and die (Sunquist, Karanth & Sunquist, 1999). Other tigers will disperse to and establish territories in the gaps left by dead tigers. Animals at different age and stage classes—cub, juvenile, transient, and breeding—have distinct behaviors associated with them in the model (Karanth & Stith, 1999). Cubs (0–1 years old) and juveniles (1–2 years old) stay in their natal territory and die if their mom dies. Individuals enter the transient stage (2–3 years old) immediately following the juvenile stage and are no longer associated with their natal range. Transients therefore do not die if their mom dies; however, they do not have an explicit movement process until they reach the breeding stage. Once entering breeding stage, animals disperse in order to establish a territory.

Breeding females (3+ years old) seek to acquire a given amount of prey resources, whereas breeding males seek to acquire a given number of females (Sunquist, 1981; Smith, 1993; Ahearn et al., 2001). An adult female will add prey resources to her territory until she can maintain basal metabolic rate (number of calories an individual burns to perform basic life-sustaining functions) of 76 kg/month (Miller et al., 2014). Female territories do not overlap; however, they can adjust their size and shape to encompass the highest possible prey resources in the area. For example, a female can adjust her territory to encompass some or all of a territory left vacant by a female that recently died. Furthermore, neighboring females can occasionally compete over territorial borders but that competition rarely leads to mortality, unless a female’s territory is already so small to begin with that losing parts of her territory to other females could cause her to starve.

Dispersing males will challenge breeding males for access to his females, with the probability of the dispersing male winning based on age (Kenney et al., 2014). If he wins, he gains access to all the females of his opponent and there is a probability that any dependent cubs and juveniles of those females will die via infanticide by the new male (Pusey & Packer, 1994). Whereas, if the dispersing male loses the challenge there is a probability that he (i) dies or (ii) moves on to another location where he will continue to search for females or challenge males for access to females (Kenney et al., 2014). Breeding males that lost a challenge to a dispersing male and did not die will become dispersing males again.

### Experiments

We created two configurations for transport infrastructure in Chitwan National Park: (i) the existing road network consisting of primary and secondary roads; and (ii) the combination of the existing road network and the alignment for a proposed electrified railway through the park’s buffer zone just north of the park core. These configurations allow us to assess the effects of existing road network and its synergistic effects with the proposed railway.

**Figure 2:**
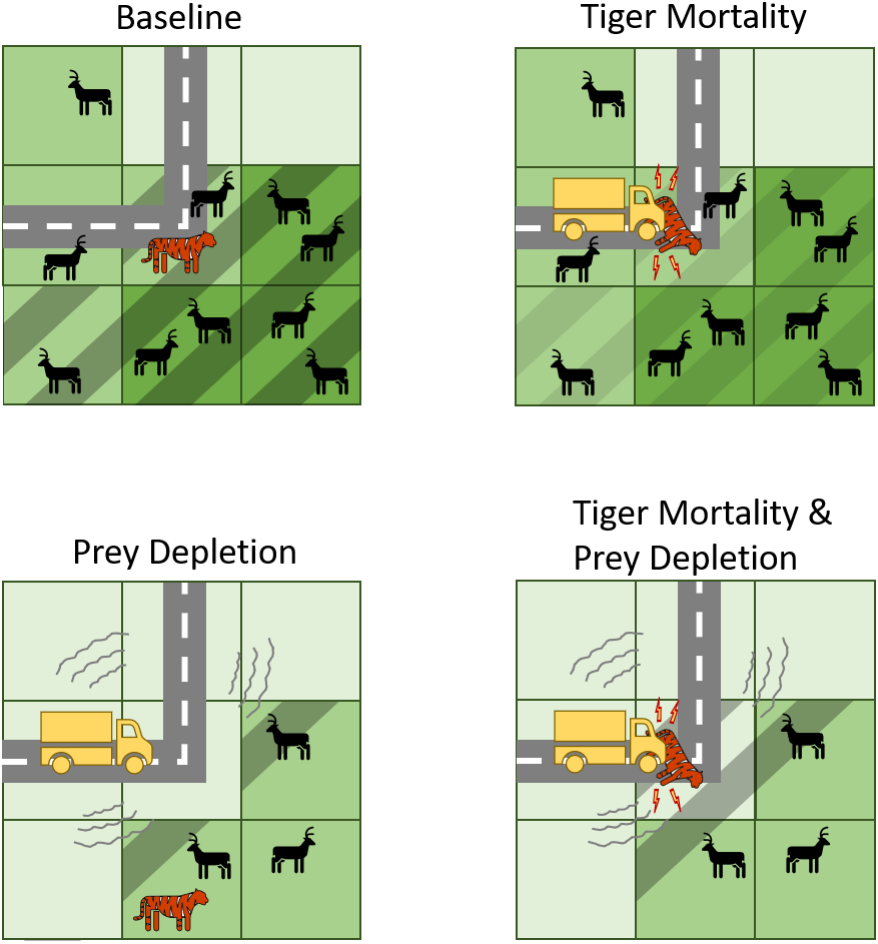
Conceptual diagram illustrating how transport infrastructure impacts tigers and their prey in the predictive model. Baseline includes the presence of roads but no effects on tiger mortality or prey abundances. Note, however, that tigers can die of natural causes while their territories intersect roads. In contrast, the other experiments include mechanisms by which transport infrastructure increase tiger mortality or deplete tiger prey, or both. Cells with dashed lines indicate those that belong to the tiger territory. A darker shade of green indicates more prey resources.

For each of the two configurations we experimentally tested the effects of different scenarios, including (i) transportation-induced increases in tiger mortality, (ii) transportation-induced depletion of prey abundance, and (iii) the interaction between increased mortality and prey loss (Fig. 2). To parameterize the *tiger mortality* experiments, we relied on previous work in Russia that found that female tiger survival declines by 67% when primary roads bisect their 50% minimum convex polygon home ranges and by 10% when bisected by a secondary road (Kerley et al., 2002). Thus, in the model, breeding females with any of their territory intersecting a primary road/railway had their annual survival rate decreased from 0.9 to 0.297 and if intersecting a secondary road their annual survival rate decreased from 0.9 to 0.81 (Fig. 2). If spanning both, then her annual survival rate was decreased by the larger of the two (*i.e.*, primary road/railway). We are not modeling the encounter rates between tiger agents and roads but rather enforcing a reduction of survival based on the presence of a road within a female’s territory. We also assumed no transportation-induced mortality of males, as we had no empirical estimates with which to parameterize that effect and we anticipate population dynamics will be strongly affected by changes in the mortality of reproducing females.

For the *prey depletion* experiments, we assumed that prey abundance was zero along a road or railway and then increased linearly as distance from the road or railway increased. For primary roads and the railway, prey was depleted up to 5 km and for secondary roads up to 2 km (Fig. 2). Prey abundances were unaffected by roads or the railway past those distances. These distances were derived from two sources. The first source was a global meta-analysis showing that mammal abundances declined up to about 5 km from roads (Benítez-López, Alkemade & Verweij, 2010). We therefore set 5 km from primary roads as the max distance at which prey are depleted. The paper indicated a non-linear effect; however, we used a linear function for simplicity and ease of interpretation, especially since the specific shape of the non-linear function is unknown for tigers and their prey. The second source was a study that calculated a relative cost of movement for tigers in areas with varying road densities (Rathore et al., 2012). Areas with medium road densities were considered 2.5 times more costly to move on than areas with low road densities, assuming that an area having a high density of roads would be avoided compared to an area with few or no roads (Rathore et al., 2012). Although not an exact proxy, we used this ratio to approximate the road effect zone for secondary roads. Thus, we set 2 km from secondary roads as the max distance at which prey are depleted.

We also tested the interaction between *tiger mortality* and *prey depletion* for each transportation configuration. These three experiments—*tiger mortality*, *prey depletion*, and *tiger mortality* and *prey depletion*—were compared against a *baseline* experiment in which roads were present but they did not affect tiger mortality rates or prey abundances. This comparison allowed us to determine how many tigers with roads/railway intersecting their territories died due to those roads/railway on top of those that would have died from natural causes such as old age or starvation in the baseline.

The models were initialized with 14 females and 7 males, numbers chosen to avoid large cycles in population size before activation of the experiments. The model runs for 600 time steps (months), approximately 15 tiger generations, without effects from roads or the railway to give the tiger population enough time to reach quasi-stationary dynamics. Then, transportation effects occur instantaneously across the landscapes at time step 601. Although instantaneous changes to prey and tiger mortality are unlikely, we assessed their effects by running the model for 240 times steps (20 years) to give the model enough time to reach a new quasi-stationary state while also being a reasonable time period. The model code implemented in the NetLogo program (Wilensky, 1999) is available online at https://github.com/nhcarter/tiger_abm.

### Data analysis

Experiments were replicated 28 times to take into account variation due to model stochasticity (Carter et al., 2015). For each experiment, we plotted through time the mean and 95% confidence intervals (based on quantiles of the t-distribution) across 28 replications of monthly population sizes of adult tigers, breeding females, and dependent offspring (*i.e.*, cubs and juveniles). We also plotted annual mortality rates along transport infrastructure as well as summed total deaths over the 20-year simulation for each experiment. This way we could examine how the experiments altered population sizes and mortality rates along both primary roads/railway and secondary roads relative to a baseline (*i.e.*, road present but no impacts on tigers or their prey). In addition, we calculated the average population size of different groups—adult tigers, breeding females, and dependent offspring—for the last time step of each of the experiments to assess how they affected tiger numbers after 20 years relative to the baseline. We used unpaired two-sample Wilcoxon tests to test for statistical differences in the means between experiments for (1) the adult tiger population sizes at the last time step and (2) total tiger deaths summed over the entire simulation. We tested differences in means within each road scenario as well as between the two scenarios.

To illustrate the spatial effects of transport infrastructure, we plotted female occupancy per cell as the proportion of all time steps (240) that any breeding female had her territory on a cell. We calculated female occupancy for all four experiments—(i) *baseline*, (ii) *tiger mortality*, (iii) *prey depletion*, and (iv) *tiger mortality* and *prey depletion*—using the existing road configuration. We ran 10 replications for each experiment to calculate mean female occupancies per cell over 20 years.

## Results

### Tiger populations

**Figure 3:**
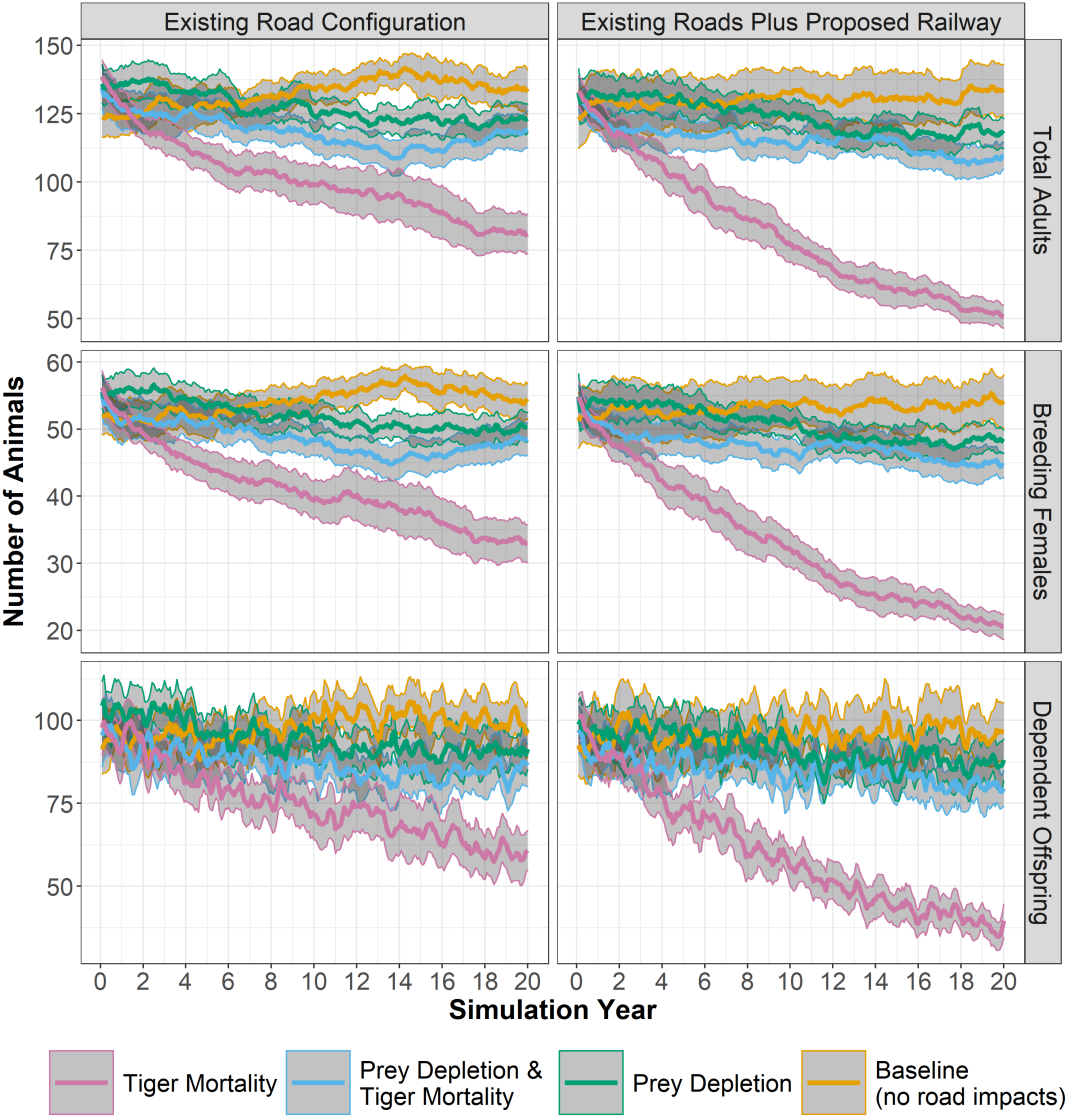
Tiger populations through time. Simulated tiger population sizes through 20 years, based on different experiments of transportation-induced impacts to tigers and their prey. Bold colored lines show mean values across 28 replicates, with confidence limits (95%) for the mean in grey. Panels at left show results for the existing road configuration and panels at right add the planned railway alignment. Number of total adults, breeding females, and dependent offspring are shown.

All three experiments—*mortality*, *prey depletion*, and *prey depletion and tiger mortality*—ended with tiger populations that were significantly lower than the *baseline* (Table 2). The population of adult tigers rapidly decreased in experiments with increased probability of mortality when tiger territories intersect primary or secondary roads. Using the existing road configuration, the average size of the adult tiger population in the *mortality* experiment decreased, after 20 years, 39% compared to the *baseline*, from 133 to 81 animals (Table 2, Fig. 3). The decrease in tiger populations was greater when adding the proposed railway: the average size of the adult tiger population in the *mortality* experiment decreased 62% (from 133 to 51 animals) compared to the *baseline*. Moreover, mortality associated with the railway alone contributed to more than 20% reduction in tiger numbers (81 to 51 individuals) over 20 years compared to the *mortality* experiment without the railway (Table 2, Fig. 3).

**Table 2:** Size of the adult tiger population at the beginning and end time steps (240 months) of simulations for different experiments. Also included are the proportional changes in population size between the starting and ending value, as well as between the ending size for each experiment compared to the baseline with no road impacts.

	**Start**	**End**	**Difference between start & end**	**Difference relative to baseline**
***Existing Road Configuration***				
Baseline (no road impacts)	123.86	132.96	0.07	0.00
Prey depletion^a^	136.00	123.29	−0.09	−0.07
Mortality^a,b,†^	138.57	81.11	−0.41	−0.39
Depletion & Mortality^a,c,‡^	134.61	119.25	−0.11	−0.10
***Existing Roads Plus Proposed Railway***				
Baseline (no road impacts)	122.82	133.86	0.09	0.01
Prey depletion^a^	132.57	117.71	−0.11	−0.11
Mortality^a,b,†^	133.25	50.75	−0.62	−0.62
Depletion & Mortality^a,c,‡^	132.57	109.57	−0.17	−0.18

**Notes.**

For each road scenario, significant differences (*p* < 0.05) between a given experiment compared to Baseline, Prey depletion, and Mortality are indicated with superscripts a, b, and c, respectively. Experiments indicated with superscripts † and ‡ differed significantly between the two road scenarios.

In contrast to the *mortality* experiments, the population of adult tigers did not respond as severely to the experiments in which prey densities are diminished near primary and secondary roads. Using the existing road configuration, the average size of the adult tiger population in the *prey depletion* experiment decreased 7% compared to the *baseline*, from 133 to 123 animals (Table 2, Fig. 3). Adding the railway decreases adult tiger numbers an additional 3% (10% total) due to loss of prey. Interestingly, combining prey depletion and tiger mortality from roads did not additively decrease tiger populations but in fact reduced the effects of considering *mortality* alone. Using the existing road configuration, the average size of the adult tiger population in the experiment combining *prey depletion and tiger mortality* from roads decreased 10% compared to the *baseline*, from 133 to 119 animals (Table 2, Fig. 3). However, adding the railway to the existing road configuration nearly doubles the loss of tigers over 20 years (133 to 110) from the combination of *prey depletion and tiger mortality* (Table 2, Fig. 3). The changes to population sizes of adult females and dependent offspring are approximately proportional to those of the total adult population.

### Patterns of mortality

**Figure 4:**
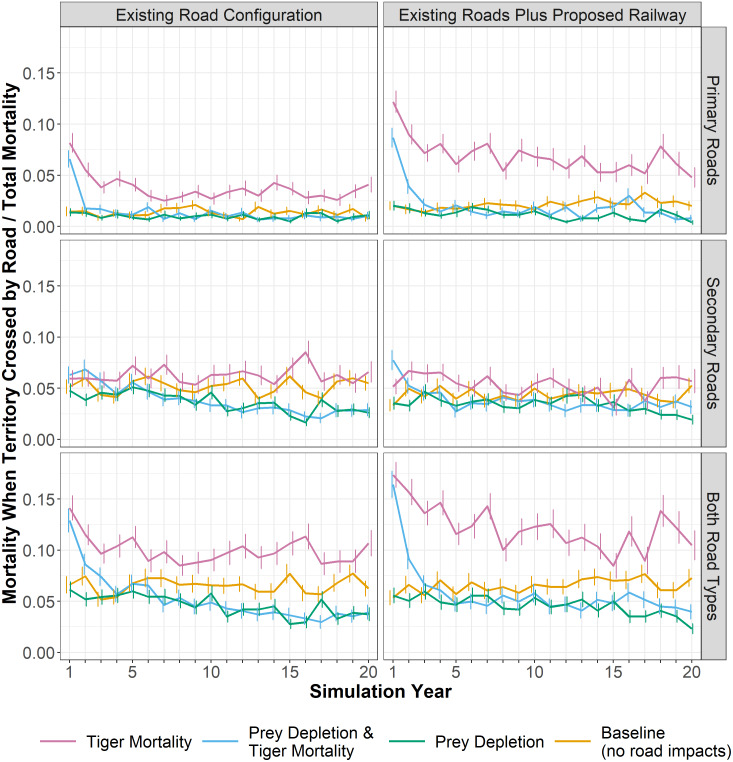
Tiger mortality and roads. The proportion of all tiger mortality attributed to roads; that is, those females that died while their territory intersected a road. Lines show an annual average, calculated as the monthly proportions averaged for each year across 28 replicates. Whiskers show the standard error of the annual averages across the 28 replicates. Panels at left show results for the existing road configuration and panels at right add the planned railway alignment. Mortality rates are shown for primary roads, secondary roads, and both road categories.

In the *mortality* experiment, the death of tigers with territories crossed by existing roads was on average 10% of all mortality (Fig. 4), compared to only 6.5% in the *baseline* (*i.e.*, tigers with territories crossed by existing roads that died from natural causes). The proposed railway increased this average proportion to 12.3%, nearly doubling the rate in the *baseline*. For most experiments, the proportion of deaths on secondary roads was approximately 2 to 3 times greater than those on primary roads/railway (Fig. 4). However, in the *mortality* experiments including the railway, a greater proportion of tigers died along primary roads (a category encompassing the railway) than on secondary roads. In the *mortality* experiments, the mortality rates are highest in the first few years, declining somewhat and stabilizing after 7 or 8 years once the population density decreased following the initial deaths of those tigers with territories crossed by roads. In the *prey depletion* experiment, the mortality of tigers intersecting roads is lower than all other experiments through time. Likewise, although higher in the first 3 to 4 years, the proportion of tiger deaths along roads or the railway becomes lower in the combined *prey depletion and tiger mortality* experiment than in the *baseline*. Prey depletion appears to dampen the effects of mortality rather than amplify it.

**Figure 5:**
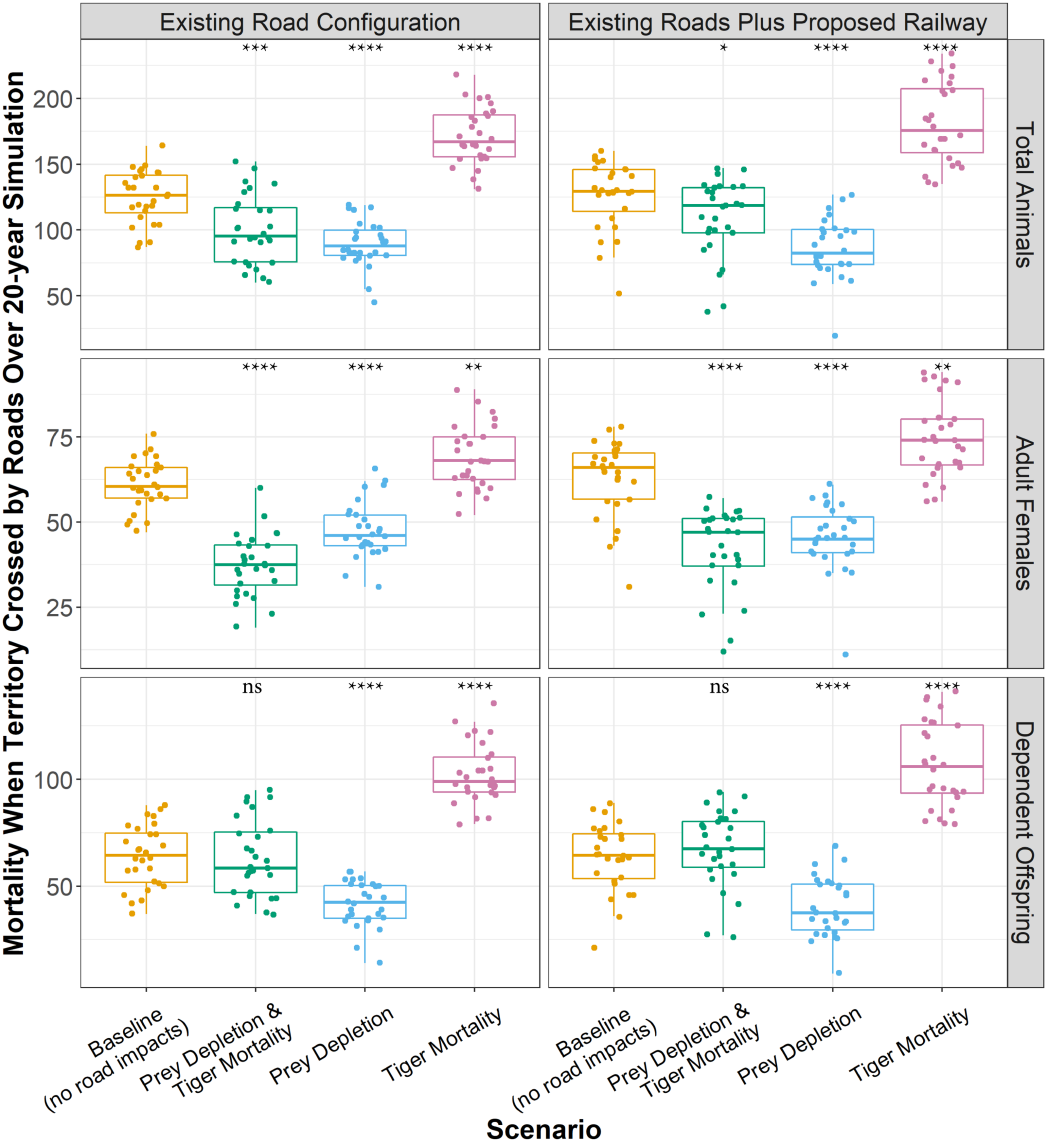
Total tiger mortalities along roads over 20 years. Boxplots showing the number of tigers that died while their territory was crossed by transport infrastructure over 20 years for each model experiment. Boxplots represent the 25th and 75th percentiles of deaths across 28 model replicates. Each replicate is shown as point. Whiskers represent the 95% confidence intervals, and black lines within boxes represent medians. The means across replicates for each experiment were statistically compared against the baseline using Wilcoxon tests. ns: *p* > 0.05, *: *p* ≤ 0.05, **: *p* ≤ 0.01, ***: *p* ≤ 0.001. ****: *p* ≤ 0.0001.

About 171 tigers with territories crossed by roads died over 20 years in the *mortality* experiment (Fig. 5), which was 46 more than those that died in the *baseline* indicating those additional deaths were due to the increased likelihood of mortality caused by roads. The proposed railway directly added about 10 more tiger deaths over 20 years (Fig. 5). In the *baseline*, the proportion of adult females and dependent offspring that died were approximately equal. The same was true of the *prey depletion* experiment, although a few more adult females died than dependent offspring. Furthermore, the total numbers of adult females and dependent offspring that died over the 20-year simulation were significantly less in the *prey depletion* experiment than in the *baseline*. In contrast, in the *mortality* experiments, the proportion of dependent offspring that died along transport infrastructure was greater than the proportion of adult females that died along transport infrastructure. However, the causes of those disparities are different. In the *mortality* experiments, existing roads and the proposed railway are causing more adult females and dependent offspring to die than those that died from natural causes in the *baseline* (13–19% more), with greater impacts on dependent offspring (60–68% more, Fig. 5). In the combined *prey depletion and tiger mortality* experiments, 24 fewer adult females die than in the *baseline*, whereas approximately the same number of dependent offspring die (Fig. 5). That is, the chances of adult females with territories crossed by roads dying are lower than those in the *baseline*.

### Patterns of occupancy

The average occupancy per cell, measured as proportion of timesteps occupied by a female tiger, was 0.64 for the *baseline*. In other words, on average, a cell in the model was occupied by a female tiger for about 13 out of the 20 years in the simulation. The average occupancy for the *mortality* experiment was 0.4, nearly 40% lower than the *baseline* and equivalent to only 8 out of the 20 years. Experiments that depleted prey were less severe. In the combined *prey depletion and tiger mortality* experiment, the average occupancy was approximately 15% lower (0.55) than the *baseline*. The *prey depletion* experiment had little effect, overall, on average occupancy, which was about 4% (0.61) lower than the *baseline*.

**Figure 6:**
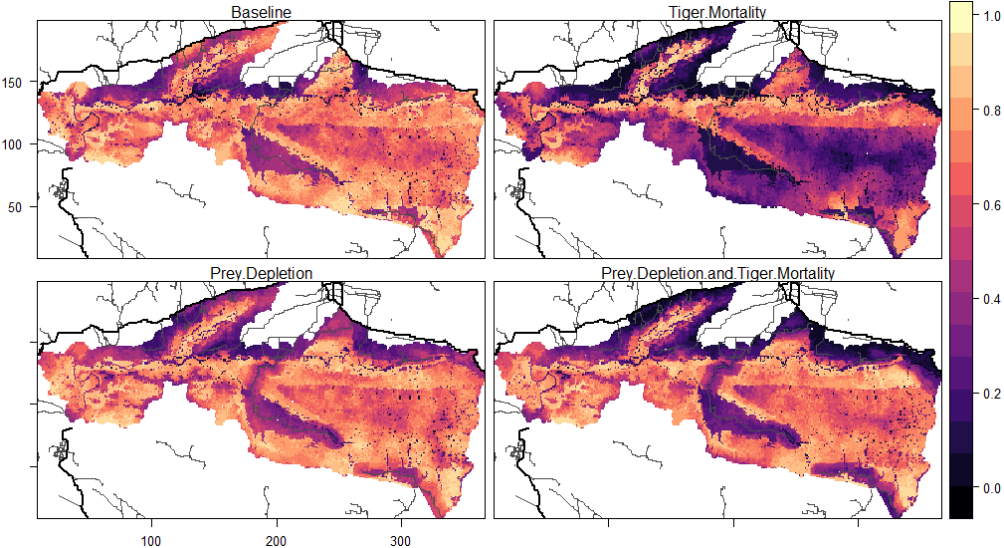
Patterns of tiger occupancy for each model experiment. Values show the proportion of the 240 time steps in which a cell was occupied by an adult female tiger, averaged across 10 replicates. Darker colors represent lower values and brighter colors represent higher values. Primary roads are shown as thick black lines and secondary roads as thin black lines. The railway was not included in this analysis. The *X*, *Y* dimensions are model coordinates not geographic coordinates.

The locations of roads affected tiger occupancy patterns and differences between experiments. Here, occupancy is defined as the proportion of months within 20 years in which a grid cell was part of a female tiger territory. Female occupancy was lower in cells near roads (*i.e.*, 2 km from secondary roads and 5 km from primary roads, 0.57) than far from roads (0.68) in the *baseline* (Table 3). The *baseline* pattern of female occupancy corresponds to the distribution of prey across the landscape, where the mean prey resources of cells near roads was 2.89 and 4.3 far from roads. Compared to the *baseline*, *mortality* experiments equally decreased female occupancy (∼1/4 of total) in cells near (0.57 to 0.32) and far (0.68 to 0.44) from roads (Table 3, Fig. 6). That is, road-induced mortality decreased the average female occupancy across the entire landscape by 5 years in the 20-year simulation. In contrast, reductions in female occupancy for experiments that depleted prey largely concentrated the changes near roads, compared to the *baseline* (Fig. 6). The *prey depletion* experiment did not substantially change female occupancy patterns far from roads compared to the *baseline*, but decreased the occupancy by nearly 20% in cells near the roads (Table 3). The reduction in female occupancy near roads compared to the *baseline* was even greater (40%) in the combined *prey depletion and tiger mortality* experiment (Table 3, Fig. 6). In this experiment, tigers only occupy cells near roads about 6.6 years of the 20-year simulation, compared to 11.4 years in the *baseline*.

**Table 3:** Female tiger occupancy, *i.e.*, the average proportion of 240 time steps (months) in which cells near or far from roads were occupied by a breeding (territory-holding) female tiger for each experiment. Areas near roads included cells that were 2 km from a secondary road or 5 km from a primary road. All other cells were considered far from roads.

**Experiment**	**Near roads**	**Far from roads**
Baseline (no road impacts)	0.57	0.68
Tiger Mortality	0.32	0.44
Prey Depletion	0.46	0.70
Depletion & Mortality	0.33	0.68

## Discussion

We found that direct tiger mortality caused by existing roads in Nepal’s Chitwan National Park would cause dramatic reductions in tiger numbers in just 20 years. Existing roads killed an additional 46 individuals over 20 years and reduced the population by 39%, compared to the counterfactual wherein road-induced mortality was absent. Occupancy results indicated that consistent road mortality more frequently created vacant tiger territories that were filled by animals from all over Chitwan, akin to compensatory immigration (Cooley et al., 2009). In effect, road mortality suppressed tiger occupancy across much of Chitwan, even in areas far from roads.

Tiger numbers were therefore highly sensitive to increased mortality associated with transport infrastructure, consistent with other simulation models of large carnivores. For example, a model of a jaguar (*Panthera onca*) metapopulation in Brazil estimated that road mortality would cause the metapopulation to decrease 36% from 351 to 226 animals after 100 years (Cullen Jr et al., 2016). The decrease was nearly 80% (351 to 73 animals) when doubling the road mortality rate, significantly increasing the extinction risk of the metapopulation (Cullen Jr et al., 2016). In our simulations, about 10% of total tiger mortality was caused by existing roads, which roughly corresponds to empirical estimates on tigers and other large carnivores. For example, previous work in Russia indicated that vehicle collisions accounted for over 8% of tiger mortality (Goodrich et al., 2008). Given higher tiger densities in Nepal than in Russia, it is likely that a higher proportion of tigers would encounter roads and thus die by vehicle collisions than in Russia. Another study found that vehicle-related mortalities of the Florida panther (*Puma concolor coryi*) accounted for 20% of all mortalities over a 25-year period from 1981 to 2004 (Schwab & Zandbergen, 2011). Thus, our simulated rates of transportation-induced tiger mortality are realistic and portend significant consequences on tiger populations if not mitigated.

Based on existing parameters, we assumed in our model that transportation-induced mortality only directly affected adult females and any dependent offspring they may have. Because breeding females drive population growth, populations in which breeding females experience elevated mortality from roads and railways will be at greater extinction risk. Disproportionate effects of roads on females of other large carnivores have also been documented. In a study collating leopard (*Panthera pardus*) survival data in southern Africa, 6 of the 20 (30%) adult female leopards living outside protected areas were killed by vehicle accidents on roads (Swanepoel et al., 2015). Whereas, only 1 of 15 (6.7%) adult males living outside protected areas were killed by vehicle accidents on roads (Vickers et al., 2015; Swanepoel et al., 2015; Mohammadi et al., 2018). In contrast, Naderi, Farashi & Erdi (2018) found that road-induced deaths of male Persian leopards (*Panthera pardus saxicolor*) were about 2.5 times more common than females, because males dispersed more widely than females and consequently encountered roads more often. Likewise, on average, male tigers disperse farther than females, putting them at risk from vehicle collisions (Smith, 1993). Our model may therefore underestimate population declines as we did not include road-induced changes to adult male mortality. Another important source of uncertainty involves mortality of dependent offspring. In the model, there were increased deaths of cubs and juveniles whose mothers were killed by roads. Although dependent offspring might survive to adulthood following the death of their mother, empirical evidence suggests that the fate of cubs is tied closely to their mothers (Kerley et al., 2003). Increased mortality of dependent offspring reduces the number of animals reaching adulthood and therefore decreases opportunities for future breeding. The high sensitivity of tiger populations to increased mortality caused by transport infrastructure indicates the importance of having robust estimates for those parameters. Those parameters are sparse across tiger range, except for some data in Russia. We recommend field research be conducted to quantify transportation-induced mortality rates for different age-classes, sexes, seasons, and habitats to improve predictions and mitigation efforts.

We found that tiger mortality associated with the proposed railway caused an outsized impact on the population, despite the railway cutting through a relatively small portion of Chitwan National Park’s buffer zone (*i.e.*, Barandabhar corridor). The proposed railway placement directly killed 10 more tigers over 20 years than those that died along existing roads; however, those rail-induced deaths were ramified through the population, leading to the removal of an additional 30 adult tigers from the population (81 to 51, Table 2). That is, fewer females mean fewer litters which depresses the population size even more. Because the proposed railway site passes just south of the existing East-West highway through a narrow forest corridor, the railway and nearby highway may be causing an ecological trap. Tigers settling in that region are almost certain to die from the highway or the railway, as tiger territories are constrained within that small forest fragment. Furthermore, the proposed railway passes through an area with high densities of tigers and their prey (DNPWC, 2018). Tigers in the model will therefore be drawn to areas near the railway, putting them at greater likelihood of dying.

We assumed that the railway would decrease tiger survival to the same degree as a primary road; however, empirical data on rail-induced tiger mortality caused by collisions with trains are limited. A recent news report indicated that five tigers (and seven leopards (*Panthera pardus*)) had been killed in the last 5 years by trains along a 20-km railway passing through India’s Ratapani Tiger Reserve (Tomar, 2021). There might be a lower likelihood of tiger mortality through collisions with trains than collisions with vehicles on primary roads, because traffic volume is typically lower on railways than primary roads. It is also possible that tiger exposure to railways is much lower than to roads, as roads are far more common in tiger range. Yet, research has shown that railways pose a negative, underexplored effect on some carnivores, particularly if food like grain spills out of the train cars which can attract prey and consequently predators (St. Clair et al., 2017; Tomar, 2021). Railways are also known to restrict tiger and other animal movements (Yumnam et al., 2014; Dutta et al., 2016). In the case of Chitwan that would mean the proposed railway would cut off tigers from important forests in the north, blocking one of the few corridors available to the isolated Chitwan population. Future iterations of the model would explicitly incorporate changes to connectivity caused by transport infrastructure on population dynamics. Furthermore, combining our ABM with empirically-informed statistical models, such as hidden Markov models and hierarchical Bayesian models, could help tease apart the relative contribution of varying sources of mortality to tiger extinction risk (McClintock et al., 2020).

Contrary to our expectation, experiments in which roads caused prey depletion considerably dampened the effects of transportation-induced tiger mortality. The reason for that result is that depletion of prey also decreased tiger use and occupancy of sites close to roads, as tigers shift away from locations with low prey. Therefore, prey depletion near roads reduces tiger exposure to transportation-induced mortality. Our occupancy results show that the *prey depletion* experiment decreased the overall prey and occupancy of tigers near roads, whereas the effects on prey and occupancy were minimal farther from roads. Empirical studies have demonstrated similar patterns. In Chitwan, Nepal, Bhattarai & Kindlmann (2013) found that the likelihood of encountering tiger prey species decreased with increasing human disturbance, which included distance to roads. In Ecuador, the biomass of prey species for jaguar was 2-4.5 times greater at the sites with lowest human access, defined in part by road density, compared to the high access sites (Espinosa, Celis & Branch, 2018). Predators follow similar patterns. In China, tigers were five times more likely to occupy areas at least 4 kilometers away from roads than they were to be found near roads (Wang et al., 2018). Jaguar occupancy in Ecuador was up to 18 times higher in the most remote site than the most accessible site (Espinosa, Celis & Branch, 2018).

Although the depletion of prey near roads may at first indicate that tiger road mortality is of a much lesser concern, there are many other implications of prey reduction that have to be considered. First, the depletion of herbivores constitutes a major conservation challenge in itself, for example, by detrimentally affecting ecosystem functioning and reducing carrying capacities for tigers and other predators (Jia et al., 2018). Furthermore, the changes to space use patterns of tigers and their prey caused by roads and railways might alter human-wildlife dynamics in nearby human communities. Herbivorous prey displaced by roads may forage more frequently in crop fields or tigers may hunt livestock more often. Indeed, attacks on people by tigers from 2010 to 2014 was found to strongly relate to forest fragmentation (Acharya et al., 2017). Interestingly, a spate of tiger attacks on people has occurred along the East-West highway in Nepal’s Bardia National Park, begging the question of whether the road itself has contributed to these attacks by fragmenting the landscape. The results of our model constitute a strong argument for taking into account prey distributions into the planning of roads and railways. Placing roads or railways in locations with currently low prey densities might reduce the risk of road mortality for both tigers and their prey. Incorporating prey dynamics, such as from predation pressure and changing seasons, will enable future versions of the model to assess the temporal dynamics in predator–prey interactions under different scenarios of change.

Tiger numbers have increased in Nepal from 2009 to 2018; however, the mortality rate has increased as well (Bhandari, Shrestha & Aryal, 2019). Bhandari, Shrestha & Aryal (2019) found that these deaths were caused by fighting, natural causes, poaching, and road accidents. Notably, the tiger population in Chitwan decreased from 120 to 93 adult tigers between 2013 to 2018. The main cause of that decline in Chitwan is unclear, though the degradation of habitat quality due to anthropogenic pressures is a plausible explanation (Bhandari, Shrestha & Aryal, 2019). Thus, declining tigers in Chitwan may correspond to population trajectories in our model experiment combining transportation-induced tiger mortality and depletion of prey. There is also growing evidence that vehicle-caused deaths of tigers and their prey in Nepal have been increasing in the last few years. Only one vehicle collision with a tiger had been recorded along the East-West highway prior to 2019. However, from 2019 to 2021, three tigers have been hit in Bardia National Park and two in Parsa National Park, which adjoins the eastern side of Chitwan National Park. Between 2018 and 2019, 45 of 67 wild animal deaths—including key tiger prey like sambar deer—were from traffic accidents in Banke National Park. Transportation-related deaths of tigers and their prey might continue to increase, given future development trends in Nepal. The Government of Nepal plans to add more than 20,000 km of transport infrastructure by 2030, including the construction of more provincial and national highways, and railways across the country (NPC, 2020).

We urge decision makers in Nepal to make sustainable transportation development a top priority to alleviate its detrimental impacts to tigers. Numerous options exist including smart green infrastructure planning that promotes tiger-friendly projects: for example, avoiding critical tiger habitats (like Chitwan), using wildlife crossings to reduce tiger mortality and facilitate habitat connectivity between populations, and compensating for damages to tiger habitats caused by infrastructure development to ensure net positive impacts (Quintero et al., 2010). An important first step is to prohibit transportation development from “no go” zones that encompass tiger source populations. Such a “no go” zone should include Chitwan National Park and adjoining forests, which harbor the largest tiger source population in Nepal. Likewise, we recommend alternative placement of the proposed railway such that it would avoid the national park and its buffer zone, as our model indicates the planned alignment through Barandabhar forest would negatively affect the future of tigers in Chitwan. Railway mitigation techniques such as reducing track curvature and train speed in tiger habitats can also reduce animal mortality by giving animals more time to detect oncoming trains (St. Clair et al., 2020). On roads, reducing traffic volume and speed can substantially reduce the likelihood of wildlife-vehicle collisions (Glista, De Vault & De Woody, 2009). In Nepal’s Bardia National Park, guard posts enforce speed limits on motorists traveling on the East-West highway that passes through the park. To our knowledge, speed limits are not enforced through the Chitwan-Parsa complex yet doing so would likely reduce the impact of vehicles on wildlife in that critical habitat. Other measures that could be tested include reducing traffic volume during nighttime when many wildlife species are most active and when motorists have lower visibility than the daytime. Moving forward, models like ours can be integrated into structured decision making, whereby the benefits and costs of transport infrastructure to human societies and biodiversity can be evaluated before the land is permanently transformed by roads and railways (Laurance et al., 2014).

## Conclusion

Transport infrastructure is encroaching the habitats of many endangered species globally. Yet, research on the potential impacts of proposed roads and railways on populations of these animals is critically limited. Without this information, planned infrastructure projects may severely jeopardize wildlife conservation efforts. We spatially forecasted the effects of transport infrastructure on a population of globally endangered tigers via two main mechanisms—transportation-induced tiger mortality and depletion of prey. We found that transport infrastructure can have strong negative effects on tiger populations over time if it increases tiger mortality, though the depletion of prey near roads and the railway buffered these effects. Overall, the spatial interaction between transport infrastructure, prey abundances, and tiger territories was an important driver of tiger population dynamics over time. Using models like ours, conservation and land-use planners can develop smart infrastructure that explicitly accounts for and mitigates the impacts of transport infrastructure on endangered wildlife populations.

## Supplemental Information

10.7717/peerj.13472/supp-1 Supplemental Information 1 Data, model outputs and R code All data on tiger populations and occupancies across all scenarios and the R code used to produce all results, tables, and figures.

## Additional Information and Declarations

### Data Availability

The following information was supplied regarding data availability: The simulation outputs are available in the Supplementary File.
